# Requirements of Health Data Management Systems for Biomedical Care and Research: Scoping Review

**DOI:** 10.2196/17508

**Published:** 2020-07-07

**Authors:** Leila Ismail, Huned Materwala, Achim P Karduck, Abdu Adem

**Affiliations:** 1 Department of Computer Science and Software Engineering College of Information Technology United Arab Emirates University Al Ain, Abu Dhabi United Arab Emirates; 2 Faculty of Informatics Furtwangen University Furtwangen Germany; 3 College of Medicine and Health Sciences United Arab Emirates University Al Ain, Abu Dhabi United Arab Emirates

**Keywords:** big data, blockchain, data analytics, eHealth, electronic medical records, health care, health information management, Internet of Things, medical research, mHealth

## Abstract

**Background:**

Over the last century, disruptive incidents in the fields of clinical and biomedical research have yielded a tremendous change in health data management systems. This is due to a number of breakthroughs in the medical field and the need for big data analytics and the Internet of Things (IoT) to be incorporated in a real-time smart health information management system. In addition, the requirements of patient care have evolved over time, allowing for more accurate prognoses and diagnoses. In this paper, we discuss the temporal evolution of health data management systems and capture the requirements that led to the development of a given system over a certain period of time. Consequently, we provide insights into those systems and give suggestions and research directions on how they can be improved for a better health care system.

**Objective:**

This study aimed to show that there is a need for a secure and efficient health data management system that will allow physicians and patients to update decentralized medical records and to analyze the medical data for supporting more precise diagnoses, prognoses, and public insights. Limitations of existing health data management systems were analyzed.

**Methods:**

To study the evolution and requirements of health data management systems over the years, a search was conducted to obtain research articles and information on medical lawsuits, health regulations, and acts. These materials were obtained from the Institute of Electrical and Electronics Engineers, the Association for Computing Machinery, Elsevier, MEDLINE, PubMed, Scopus, and Web of Science databases.

**Results:**

Health data management systems have undergone a disruptive transformation over the years from paper to computer, web, cloud, IoT, big data analytics, and finally to blockchain. The requirements of a health data management system revealed from the evolving definitions of medical records and their management are (1) medical record data, (2) real-time data access, (3) patient participation, (4) data sharing, (5) data security, (6) patient identity privacy, and (7) public insights. This paper reviewed health data management systems based on these 7 requirements across studies conducted over the years. To our knowledge, this is the first analysis of the temporal evolution of health data management systems giving insights into the system requirements for better health care.

**Conclusions:**

There is a need for a comprehensive real-time health data management system that allows physicians, patients, and external users to input their medical and lifestyle data into the system. The incorporation of big data analytics will aid in better prognosis or diagnosis of the diseases and the prediction of diseases. The prediction results will help in the development of an effective prevention plan.

## Introduction

The notion of health data management systems has evolved during the last century. With the evolution of medical records from paper charts to electronic health records (EHRs) [1], health data management has undergone disruptive transitions to provide more accurate and better patient care and make qualitative use of these records. This shift is underpinned by the advancement in information technologies that led to the development of several notions of health data management systems. Those health data management systems were often misaligned with the goals of biomedical care and research. This misalignment is caused particularly by the discrepancies between advanced technologies and their adoption for biomedical care and research. Consequently, it becomes vital to address this gap by developing a new framework for the health data management system. In this paper, we provide a broader history and evolution of health data management systems underpinned by the changing definition of medical records, discuss the issues prevailing within, introduce the modern aspects of health data management systems supporting the growing size of medical data, and discuss insights and provide solutions aiming for a better health care ecosystem.

The introduction of EHRs has transformed the health care industry by providing more services, improving the quality of patient care, and enhancing the data access ability in real time, thereby creating a diverse set of health data management systems [2]. Our understanding of EHRs is that it provides a longitudinal view of a patient’s medical history over his or her lifetime generated by one or more health care providers or medical organizations delivering treatments to that patient. These cohesive and summarized records include the patient’s demographic and personal information, past and current diagnoses and treatments, progress notes, laboratory and radiology results, allergies, and immunizations [1]. However, an earlier form of EHRs referred to as paper charts involves written records of a patient’s diagnosis and treatments for the purpose of medical teaching. Next, the term has been revised to computer-based patient records, electronic medical records, and currently EHRs. With the advancement in technological developments and the goal to provide better and efficient health care, health data management systems have evolved from a computer-based approach to client-server–based, cloud, the Internet of Things (IoT), and finally to blockchain-based system.

With the rise of big health care data and the realization of using medical data for governance and research, it becomes necessary to integrate big data analytics within health data management systems [3]. However, this brings new challenges of data aggregation and preprocessing from multiple sources to develop insights, data security, and privacy to cope with an increasing number of data breaches and hacking incidents [4]. Further challenges have been imposed on biomedical care and research by the nature and types of medical data being generated at a rapid pace. These challenges have developed the need for a new health data management framework.

This paper analyzes the requirements for better patient care and predictive analysis that must be considered when implementing a health data management system. Considering these requirements will make the health care data management system more accurate, efficient, and cost-effective. To our knowledge, this is the first analysis of the temporal evolution of health data management systems to give insights into the system requirements for better health care.

The contributions of this paper are three-fold. First, the paper provides a taxonomy of health data management systems based on their technological advancement, and the inherent challenges and issues are discussed therein. Second, we present the reforming definitions of medical records and extract the requirements of a health data management system. Third, the paper provides insights into the health data management system research and guidelines for the future research area.

### Related Works

Health data management systems are evolving for better health care. Literature reviews on these systems are classified into 2 categories: (1) electronic health (eHealth) [5-8] and (2) mobile health (mHealth) [9].

Regarding eHealth, the study by Jamal et al [5] reviews the impact of a computerized system on the quality of health care. The results showed that a health information system, if properly designed, can prevent medical errors and can support doctors and medical providers in diagnosis. The study by Van De Belt et al [6] reviews the definitions of health and medicine over 2 years (from 2007 to 2009), coming up with a common definition involving the web, patients, professionals, social networking, health information content, and collaboration. In this study, we reveal additional requirements needed for better health care: privacy, security, public insights, and patient participation in accessing and monitoring medical data. The studies by Hans et al [7] and Cunningham et al [8] focus on the definitions of eHealth from 1999 to 2004. The authors found that the themes health and technologies are most recurrent in all definitions.

Concerning mHealth, Silva et al [9] provide a review of mHealth apps and services. It highlights that the coordination, integration, and interoperability between different mHealth apps is important for better health care as well as improved performance of mobile devices in terms of device battery, storage, computation, and network.

In this study, we reviewed health data management systems based on the following 7 requirements across studies conducted over the years: (1) medical record data, (2) real-time data access, (3) patient participation, (4) data sharing, (5) data security, (6) patient identity privacy, and (7) public insights.

## Methods

For the analysis and study of the evolution of health data management systems, we reviewed published research articles, reports, medical lawsuits, and health care regulations; acts about the methods of organizing medical record data; and the needs of a health data management system. The literature was searched in the Institute of Electrical and Electronics Engineers, Association for Computing Machinery, Elsevier, MEDLINE, PubMed, Scopus, and Web of Science databases from 1793 to 2020. We selected the papers that included incidents that involved the definitions of a health data management system, triggered the introduction of a new system, and/or implemented technologies for better health care. The analysis of these papers shows that advances in technologies are being adopted for accurate and efficient patient care.

## Results

### Taxonomy of Health Data Management Systems

**Figure 1:**
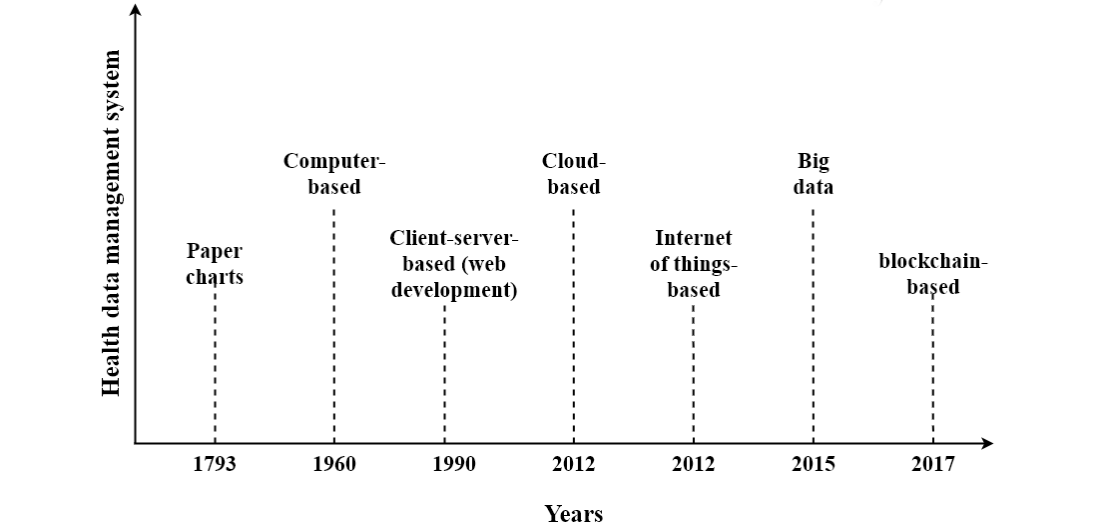
Evolution of the health data management system.

Before satisfying the requirements of biomedical care and research, the evolution of the underlying health data management systems and their limitations must be understood. The capabilities of the health data management should ensure that the requirements of patient care are met. Health data management systems have undergone multiple transitions over the years alongside the advancement in information technologies as shown in Figure 1. During this evolution, several programs were established and regulation acts were passed to improve the quality of patient care. Table 1 presents the events that triggered the evolution of health data management systems. Table 2 presents the limitations of health data management systems.

**Table 1:** Events that have triggered the evolution of health data management systems.

Year	Responsible authority	Evolutionary change
1793	Board of Governors of the Society of the New York Hospital	Rule to record patients’ data for hospital expenditure justification was passed [10].
1805	Board of Governors of the Society of the New York Hospital	Rule to record major medical cases for education was passed provoked by a fatal dispute between an American statesman and an American politician. According to the rule, the recorded cases should be bounded in a book for inspection by the governors, medical professionals and students, and the friends of the patients [11].
1830	Board of Governors of the Society of the New York Hospital	Rule to maintain a record of all the medical cases [11].
1918	American College of Surgeons	A hospital standardization program was established to standardize the format of medical records for improved patient care [12].
1928	American College of Surgeons	American Association of Record Librarians of North America was established to enhance the standards of medical records [13].
The 1960s	Lawrence Weed	The idea to use computers for medical records was proposed to allow doctors to track a patient’s medical history and provide evidence for the treatment [14]. A problem-oriented medical records model was developed to standardize the method of EMRs^a^ that provided a structure to help doctors record their notes [14].
The 1960s	N/A^b^	Paper charts were termed as EMRs.
1965	Centers for Medicare and Medicaid Services	Rule to record patients’ data by medical nurses for medical insurance reimbursement with the introduction of Medicare and Medicaid laws [15].
1965	Lockheed Corporation	First commercial computerized health data management system known as Clinical Information System was developed for El Camino Hospital. The system included features for laboratory tests, appointment scheduling, and pharmacy management [16].
1967	University of Utah, 3M and Latter-Day Saints Hospital	First clinical decision support system known as Health Evaluation of Logical Processing was developed to support clinical operations. The system helped doctors to identify cardiac contraction based on a patient’s test results’ analysis and to select an appropriate medication for infectious disease cases [17].
1968	Massachusetts General Hospital and Harvard University	The first modular computer-based health data management system known as Computer Stored Ambulatory Record was implemented. The system accommodated clinical vocabularies through clinical mapping to recognize different terms used for the same disease [18].
The 1980s	Indian Health Service	MPI^c^ was introduced to keep track of patients’ medical data to reduce unnecessary testing and adverse drug effects [19].
1987	Health Level Seven	Electronic standards were developed to address the standardization issues of health data management system development and adaption. The standards allowed the use of components from different vendors in a health data management system [20].
1991	Institute of Medicine	The term computer-based patient records was introduced in a report studying the benefits of electronic management of health records [21].
1996	US Congress	The Health Insurance Portability and Accountability Act was passed to safeguard patients’ medical records by involving role-based access control, automatic data backup, audit trails, and data encryption [22].
1999	John Mitchell	The term eHealth^d^ was coined that refers to the integration of electronic communication and information technologies for electronic transmission, storage, and retrieval of medical records both locally and remotely [23].
2000	S Laxminarayan and Robert SH Istepanian	The term mHealth^e^ was coined that refers to wireless telemedicine using mobile telecommunications and multimedia technologies for the new mobile health care system [24].
2001	Gunther Eysenbach	The definition of eHealth was expanded by incorporating business and public health to health services and defining the outcomes and stakeholders of eHealth [25].
2004	Stephen S Intille	The term uHealth^f^ was coined that refers to the use of biometric sensors and medical devices to monitor and improve a patient’s medical health [26].
The 2000s	Health care organizations	Proposed a formal definition of the term personal health records that allows patients to access their medical history and to manage it by making part of it available to selected participants by defining access control rights [27].
2003	Institute of Medicine	The term electronic health records [28] was coined.
2006	Commonwealth of Massachusetts	Massachusetts health care reform law was passed that mandated for residents to have minimum medical insurance coverage and for employers with more than 10 full-time employees to provide medical insurance coverage [29].
2006	Elliott Fisher	The term Accountable Care Organizations was coined that refers to a group of doctors, hospitals, and other health care providers who volunteer to give high-quality care to their patients to avoid unnecessary duplication of services and reduce medical errors [30].
2009	US Department of Justice, Office of Inspector General, and Human and Health Services	The Health care Fraud Prevention and Enforcement Action was established to strengthen the existing programs to prevent and reduce Medicare and Medicaid frauds [31].
2010	US President Barack Obama	The Patient Protection and Affordable Care Act was signed into law with an objective to provide an expansion of medical insurance coverage [32].

^a^EMR: electronic medical record.

^b^N/A: not applicable.

^c^MPI: master patient index.

^d^eHealth: electronic health.

^e^mHealth: mobile health.

^f^uHealth: ubiquitous health

**Table 2:** Limitations of health data management systems.

Health data management system	Limitation
Paper charts	Illegible handwriting resulting in incorrect treatments [33] and deaths [34,35]. Requires physical storage and are susceptible to unplanned destruction such as flood, fire, rodents, and degradation. Physically cumbersome to read, understand, and search for specific information. The cost and time required for paper charts to be requested for duplication and then delivered are unacceptably high.
Computer-based	Medical records are managed by the physicians and cannot be accessed by the patients. Physicians visiting a patient have to note down or memorize the patient’s medical data to return to the hospital and record it digitally, which may lead to error.
Client-server-based	A patient has no traceability on how his or her data are used. The issues of security, privacy, and single point of failure. In addition, a cohesive view of a patient’s medical data from multiple hospitals is difficult. Requires repeating medical tests at times, which results in more time, cost, and effect on health conditions.
Cloud-based	Single point of failure, loss of data control and stewardship, a requirement of steady internet connection, and data reliability [36,37].
IoT^a^-based	Data security and patient privacy are a major concern.
Big-data–based	The process of data aggregation from different storage sites is time consuming, complex, and expensive. The data are stored using different formats and requires preprocessing. In addition, preserving the security of the data and privacy of the patient identity while maintaining the usefulness of data for analysis and studies is quite challenging.
Blockchain-based	The process of ledger update on multiple nodes is energy consuming [38] and suffers from the issue of low throughput [39].

^a^IoT: Internet of Things.

### Requirements of a Health Data Management System

**Figure 2:**
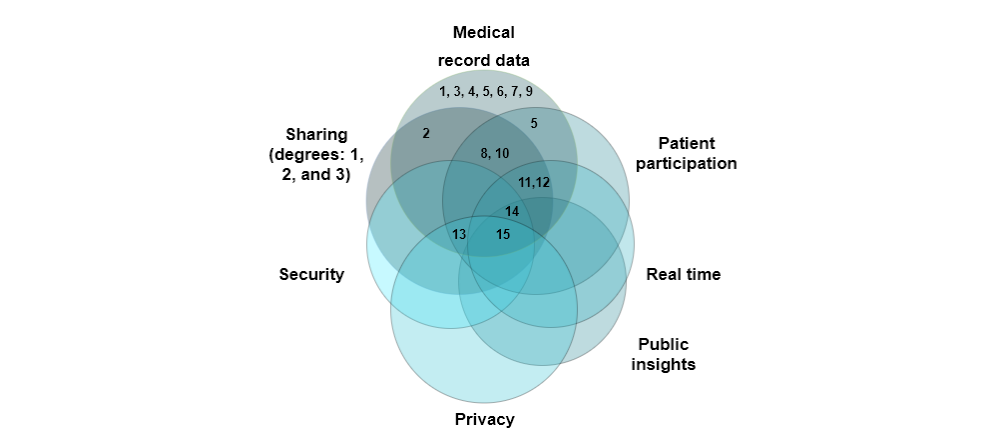
Requirements of a health data management system.

Over the last century, the definition of health data management has undergone numerous reformations to address the need for better and advanced patient care alongside technological advances. We evaluated these differing examinations and rationalized the definition used in the remainder of the paper. It is important to note that, as the term health data management is rather recent, the listed definitions were taken from different legislations and health data recording systems, even if the exact phrase *health data management* was not used. Table 3 shows the evolving definitions of health data management systems from being purely medical practice and learning-based definitions to being more patient-centric and research-based definitions. We classified health data management systems based on 7 requirements that underpin the evolution in the field as shown in Figure 2. Each number in the figure represents a definition stated in Table 3.

**Table 3:** The definitions of health data management systems.

Number	Year	Source	Definition
1	1793	Siegler [10]	“[...] Names and Diseases of the Persons, received, deceased or discharged in the same, with the date of each event, and the Place from whence the Patients last came [...]”
2	1805	Siegler [10]	“The house physician, with the aid of his assistant, under the direction of the attending physician, shall keep a register of all medical cases which occur in the hospital, and which the latter shall think worthy of preservation, which book shall be neatly bound, and kept in the library for the inspection of the friends of the patients, the governors, physicians and surgeons, and the students attending the hospital.”
3	1941	Sayles and Gordon [12]	“Accurate and complete medical records [...] which includes identification data; complaint; personal and family history; history of the present illness; physical examination; special examinations such as consultations, clinical laboratory, x-ray and other examinations; provisional or working diagnosis; medical or surgical treatment; gross or microscopical pathological findings; progress notes; final diagnosis; condition on discharge; follow-up; and, in case of death, autopsy findings.”
4	1968	Weed [14]	“The computer is making a major contribution [...] the patient will gain from his physician an immediate sympathetic understanding [...] inadequate analysis by the medical profession can be avoided.”
5	1968	Weed [14]	“[...] orient data around each problem [...] complete list of all the patient's problems [...] diagnosis and all other unexpected findings or symptoms [...] The list is separated into active and inactive problems, and in this way, those of immediate importance are easily discernible [...] orders, plans, progress notes and numerical data can be recorded under the numbered and titled problem [...]”
6	1993	Cynthia [40]	“Digital versions of paper charts that contain the medical and treatment history of the patients from one practice for providers to use for diagnosis and treatment”
7	1997	Dick et al [21]	“Electronic patient record [...] support users through availability of complete and accurate data, practitioner reminders and alerts, clinical decision support systems, links to bodies of medical knowledge, and other aids.”
8	2001	Eysenbach [25]	“[…] medical informatics, public health and business, referring to health services and information delivered or enhanced through the Internet and related technologies […] an attitude, and a commitment for networked, global thinking, to improve health care locally, regionally, and worldwide by using information and communication technology.”
9	2002	Cameron and Turtle-Song [41]	“The subjective component contains information about the problem [...] objective information consists of those observations made by the counselor [...] assessment section demonstrates how [...] data are formulated, interpreted, and reflected upon, and the plan section summarized the treatment direction.”
10	2003	Markle Foundation [42]	“[…] electronic application through which individuals can access, manage and share their health information, and that of others for whom they are authorized, in a private, secure, and confidential environment.”
11	2003	HIMSS^a^ [1]	“[...] longitudinal electronic record of patient health information generated by one or more encounters [...] patient demographics, progress notes, problems, medications, vital signs, past medical history, immunizations, laboratory data and radiology reports [...] automates and streamlines the clinician's workflow. The EHRs has the ability to generate a complete record of a clinical patient encounter [...] evidence-based decision support, quality management, and outcomes reporting.”
12	2003	HIMSS [43]	“The Electronic Health Record (EHR) is a secure, real-time, point-of-care, patient-centric information resource […] decision making by providing access to patient health record information where and when they need it and by incorporating evidence-based decision support […] billing, quality management, outcomes reporting, resource planning, and public health disease surveillance and reporting.”
13	2005	AHIMA^b^ [44]	“[...] lifelong resource of health information needed by individuals to make health decisions. Individuals own and manage the information [...] is maintained in a secure and private environment, with the individual determining rights of access [...]”
14	2008	Böcking and Trojanus [45]	“Health data management […] acquiring, entering, processing, coding, outputting, retrieving, and storing of data gathered in the different areas of health care […] also embraces the validation and control of data according to legal or professional requirements.”
15	2013	HIPAA^c^ [22]	“A major goal […] to protect the privacy of individuals’ health information […] adopt new technologies to improve the quality and efficiency of patient care.”

^a^HIMSS: Healthcare Information and Management Systems Society.

^b^AHIMA: American Health Information Management Association.

^c^HIPAA: Health Insurance Portability and Accountability Act.

#### Medical Record Data

Medical record data that describes the identity and health of a patient based on the personal and demographic identity, history of the medical condition, ongoing treatment, laboratory tests, and radiology results are the common requirement of a health data management system. The medical records have been a primary component throughout the evolution of health data management systems, whether in the form of printed documents or digital records.

#### Real-Time Data

To improve the quality of patient care, the requirement of real-time data access was highlighted in the definitions of health data management systems. This requirement reduces the medical incidents owing to the delay in data updates by the physicians. However, this requirement cannot be fulfilled by the paper-based and computer-based health data management systems. This requirement was introduced with the client-server–based management system [46-52] that enables the physicians to access and update the patient medical records in real time.

#### Patient Participation

With the medical records maintained by the hospitals or third-party cloud service providers, the patients cannot track how their medical data are used. Consequently, patient participation in accessing and monitoring medical data is a key requirement to develop trust in health data management systems. In addition to data access, the participation of patients in providing health conditions and lifestyle data to the physicians will aid in better prognosis and diagnosis. The introduction of IoT-based health data management system involving sensors and medical devices that monitor a patient’s health and lifestyle conditions enables the patient to input their medical conditions to the system [53-59]. An analysis of personal health records management platforms based on users’ perception shows that a simple easy-to-use system is required for patient engagement and satisfaction [60].

#### Sharing

Sharing of medical records is a vital requirement with the patient’s treatment being spread across various health care providers. This is to aid other physicians to study the patient’s medical history for better treatment and to avoid repetition of laboratory and radiology tests. On the basis of the list of definitions in Table 3, we classified sharing based on the users allowed to access the data into 3 different categories: (1) degree 1, where the information is shared within the same medical organization where the patient is currently receiving treatment, (2) degree 2, where the information is shared with the patient, patient’s friends, and family, and (3) degree 3, where the information is shared with other medical organizations and government. The requirement of sharing is complemented by the introduction of the cloud-based health data management system [61-63]. However, to share medical record data between different health care organizations and to efficiently use the shared information, the systems should support interoperability. Interoperability can be achieved by using a standard format to store, manage, and share the medical data. There are several standard formats to store medical data and images [64]. Some of the major file formats used for medical images are Analyze [65], Neuroimaging Informatics Technology Initiative [66], Minc [67], and Digital Imaging and Communications in Medicine [68]. Health Level 7 International, standardized by the American National Standards Institute, is a health care protocol for sharing medical data [20]. It includes the rules for the integration, exchange, and management of EHRs. Wen et al [69] assessed the interoperability of eHealth systems in Taiwan for exchanging data. This is to reduce repeated medical examinations and medications for better health care. They concluded that the government should define policies to enforce interoperability.

#### Security

With increasing incidents of data breaching and phishing attacks, and the adoption of a third-party service provider, the security of the patients’ sensitive and important data is essential. Compared with 477 health data breaches reported in 2017, affecting 5,579,438 patient records in 2017, 503 breaches affecting 15,085,302 records were reported in 2018 [70]. The requirement of security is even high when patients’ medical records are handled by a cloud service provider or when medical sensors and devices are used to gather patients’ medical and lifestyle data. According to a report by Intel Security, the use of cloud services by the health care provider has reduced owing to the lack of cyber security methods implemented by the cloud service provider [71]. A report states that, on average, hospitals lose track of around 30% of their networked medical devices, making it much harder to protect against vulnerabilities [72]. More than 61% of all IoT devices and sensors on a hospital network are at high risk of cyber-attack. In recent years, blockchain technology [73,74] has gained wide popularity and has penetrated into the domain of health care to address the need for a more patient-centric supportive system for the professionals, to connect disparate systems for improved patient care, and to increase the accuracy of EHRs [75-81].

#### Privacy

The privacy requirement of a patient’s identity in a health data management system is crucial with the increasing number of medical frauds and fake medications. The privacy of the patient cannot be compromised, especially with the rise of data analytics, where the medical record data of the patients are used for analysis. The blockchain-based health data management system aims to address this issue.

#### Public Insights

**Figure 3:**
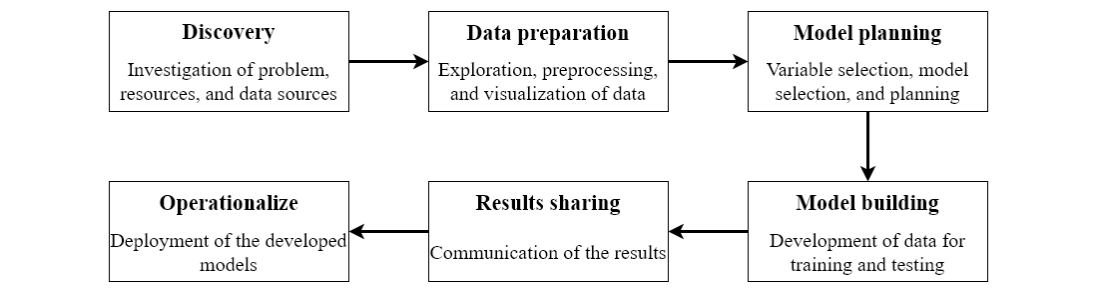
Lifecycle of big data analytics.

Prediction of health conditions is important to avoid life-threatening situations. The increasing amount of health care data [82], if properly analyzed, can facilitate the prediction of health conditions. The process of gathering, organizing, storing, and analyzing big data to discover correlations, hidden patterns, and other valuable insights is known as big data analytics. Figure 3 shows the life cycle of big data analytics.

**Figure 4:**
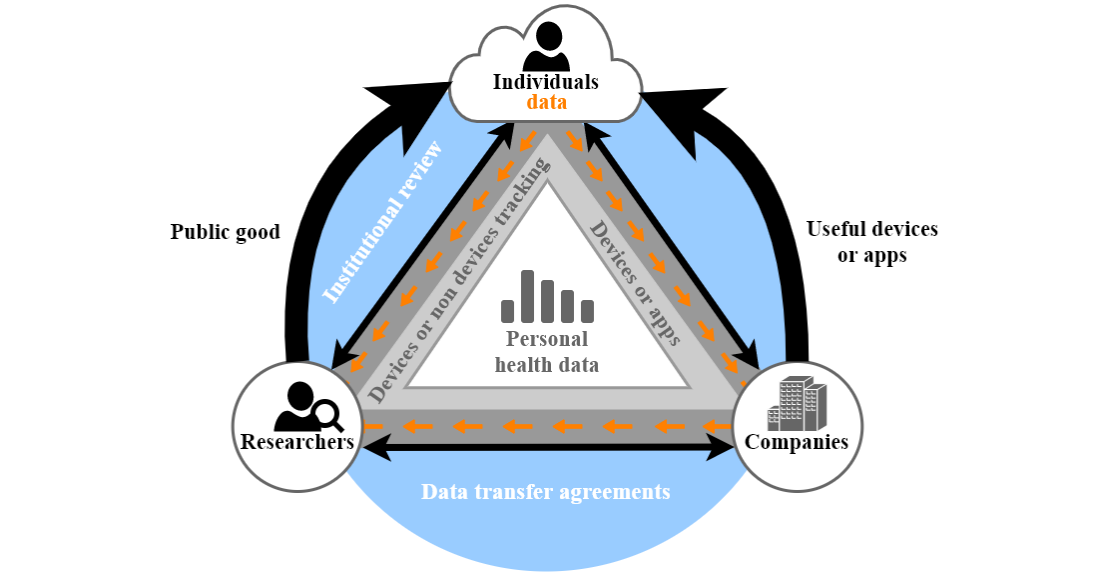
Personal health care data ecosystem.

The prediction from health care data for public insights allows to actively improve public health and to react faster to a situation [83-91]. Using personal health care data requires, of course, a well-defined balance between the assurance of the privacy of personal health care data with respect to transparency, for example, toward insurance companies. Insights into genetical personal risk factors for chronic diseases should not lead to a situation where a person has disadvantages concerning the insurance status. Moreover, the monitoring of the public health situation has to be based on the health care data of individuals. Consequently, research projects have recently addressed the balance of personal health care data as a public good [92]. Figure 4 [92] shows the relationship between the 3 key stakeholders for defining the balance between personal health care data and the potential of these data as a public good. Companies could be health insurance providers, hospitals, pharmaceutical companies, and government organizations.

The diabetes mellitus crisis or the growth of cardiovascular problems caused by nutrition patterns and lifestyle behavior in many countries and regions of the world, changing patterns of Alzheimer and dementia, or microbiome research, and the abuse of antibiotics would benefit tremendously from personal health care data as a public good [93,94]. Bringing together the insights of large initiatives such as the Health Data Exploration Project and Computational Health Sciences [92,94] promises the key for future advancement in the area of private and personal health care data for the public good. Health care data analytics can help researchers and government officials for better prediction of chronic diseases, the development of effective therapeutic drugs, more accurate patient care, and the development of a nation-wide effective prevention plan.

Table 4 shows health data management systems presented in the taxonomy and evaluates them in terms of their adherence to the defined requirements.

**Table 4:** Health data management systems in the literature vs the requirements.

System	Medical record data	Real-time data	Patient participation		Sharing				Security		Privacy		Public insights	
			Data access	Data input		Degree 1	Degree 2	Degree 3						
Paper-based	Allows recording of medical data for eventual use	Encounters high delays	Does not allow patients to track the use of their medical data	Does not allow patients to provide their health conditions		Supports data sharing only within the same hospital	Allows data sharing with the patient, patient’s friends, and family	Does not allow data sharing with the patient, patient’s friends, and family		Does not provide methods against cybersecurity attacks		Does not provide methods for preserving a patient’s privacy		Does not support prediction
Computer-based	Allows recording of medical data for eventual use	Encounters high delays	Does not allow patients to track the use of their medical data	Does not allow patients to provide their health conditions		Supports data sharing only within the same hospital	Allows data sharing with the patient, patient’s friends, and family	Allows data sharing with other medical organizations and government		Does not provide methods against cybersecurity attacks		Does not provide methods for preserving a patient’s privacy		Does not support prediction
Client-server–based	Allows recording of medical data for eventual use	Allows data retrieval in real time	Allows patients to access and monitor their medical data	Does not allow patients to provide their health conditions		Supports data sharing only within the same hospital	Allows data sharing with the patient, patient’s friends, and family	Allows data sharing with other medical organizations and government		Does not provide methods against cybersecurity attacks		Does not provide methods for preserving a patient’s privacy		Does not support prediction
Cloud-based	Allows recording of medical data for eventual use	Allows data retrieval in real time	Allows patients to access and monitor their medical data	Does not allow patients to provide their health conditions		Supports data sharing only within the same hospital	Allows data sharing with the patient, patient’s friends, and family	Allows data sharing with other medical organizations and government		Does not provide methods against cybersecurity attacks		Does not reveal a patient’s identity		Does not support prediction
IoT^a^-based	Allows recording of medical data for eventual use	Allows data retrieval in real time	Allows patients to access and monitor their medical data	Allows patients to provide health conditions		Supports data sharing only within the same hospital	Allows data sharing with the patient, patient’s friends, and family	Allows data sharing with other medical organizations and government		Does not provide methods against cybersecurity attacks		Does not provide methods for preserving a patient’s privacy		Provides methods for the prediction of health conditions
Big data analytics	Allows recording of medical data for eventual use	Allows data retrieval in real time	Allows patients to access and monitor their medical data	Allows patients to provide health conditions		Supports data sharing only within the same hospital	Allows data sharing with the patient, patient’s friends, and family	Allows data sharing with other medical organizations and government		Does not provide methods against cybersecurity attacks		Does not reveal a patient’s identity		Provides methods for the prediction of health conditions
Blockchain-based	Allows recording of medical data for eventual use	Allows data retrieval in real time	Allows patients to access and monitor their medical data	Allows patients to provide health conditions		Supports data sharing only within the same hospital	Allows data sharing with the patient, patient’s friends, and family	Allows data sharing with other medical organizations and government		Ensures the protection of medical data against cybersecurity attacks		Does not reveal a patient’s identity		Provides methods for the prediction of health conditions

^a^IoT: Internet of Things.

## Discussion

### Principal Findings

This study revealed that there is a need for a secure and efficient health data management system that will allow physicians and patients to update decentralized medical records and to analyze the medical data for supporting more precise diagnoses, prognoses, biomedical research, and public insights. The early form of health data management using the manual recording of a patient’s diagnosis and treatment on sheets of paper was introduced almost a century ago. Later, with the advancement in technology, health data management systems evolved to web, cloud, IoT, big data analytics, and blockchain-based systems. The definition of medical records has reformed alongside this temporal evolution of the system. The requirements for a health data management system extracted from these definitions are medical record data, real-time data, patient participation, sharing, security, privacy, and public insights. The paper-based health data management system fulfills the requirements of medical record data and sharing. However, paper charts are prone to misplacement, occupy large physical space, and involve a time-consuming and expensive data sharing process. Over time, the paper charts were replaced by electronic records in the computer-based system with the same requirements.

To achieve the requirement of real-time data access in addition to medical record data and sharing, a client-server–based health data management system was introduced. This system allows patients and health care providers to access medical data over the internet using a mobile device or a desktop computer. However, it suffers from the issues of single point of failure, data fragmentation, system vulnerability, low scalability, and high data security and patient privacy risks. To minimize the infrastructural cost and to address the issue of data fragmentation, the medical organizations and health care providers transitioned to a cloud-based system. The cloud service provider ensures the requirement of privacy of patient identity, but the security of the data is not ensured in addition to the issue of a single point of failure.

The requirement of patient participation to feed their medical data and lifestyle conditions for better prognosis and diagnosis was achieved with the introduction of the IoT-based management system. However, with the increasing number of data breaches and hacking of the medical sensors and devices, there prevails a constant threat to the security of data and privacy of a patient’s identity. With the advancement in big data analytics, increasing amount of health care data are being studied to gain insights for better prognosis and diagnosis of diseases. However, the privacy of a patient’s identity still remains a concern.

The blockchain technology, which recently attracted the attention of industries, shows potential in the field of health care. A blockchain-based health data management system satisfies all the requirements needed for better patient care. However, it consumes a high amount of energy [95,96] and has low throughput [39]. There are increasing research efforts to solve these issues. For instance, to address the problem of energy consumption, Milutinovic et al [97] proposed the proof of luck consensus mechanism that ensures energy-efficient and low-latency transaction validation. Ismail et al [98] and Dorri et al [99] proposed scalable blockchain architectures for health care that use a clustering approach to increase transactions throughput.

The main requirements of a health care data management system are security and privacy, especially with the increasing number of data breaching and hacking attacks. Furthermore, the adoption of patient participation to feed health data to a health system is increasing with the introduction of disruptive technologies, such as the IoT and big data analytics. Big data analytics requires the sharing of medical information among hospitals to get insights and predictive analysis from the data. This paves the way toward a health data management system as a support to physicians and medical professionals for better diagnosis and prognosis of chronic diseases. In addition, such a system allows to derive public insights from data to develop a nation-wide prevention plan for certain diseases. The traceability feature of the blockchain ensures that the data used for developing the predictive models is accurate, leading to a precise prognosis, diagnosis, and decision support system. Consequently, we suggest an integrated blockchain-, IoT- and big data–based health data management system to ensure the requirements of smart health care: real-time access to data by physicians and patients, health data input from patients through medical sensors and lifestyle, security, privacy, and public insights. This integrated health management system should be scalable and energy-efficient, presenting new research challenges in the research era of a smart health data management system.

### Conclusions

The objective of this paper was to highlight the requirements of a health data management system for biomedical care and research. In summary, it discussed the temporal evolution of health data management systems from paper charts to blockchain-based systems, along with the reformation of the definition of what we call EHRs today. The system should satisfy the requirements of medical record data, real-time access, patient participation, data sharing, data security, patient identity privacy, and public insights. The incorporation of big data analytics aids in better prognosis and diagnosis of the diseases and the prediction of risk for the development of chronic diseases.

## Abbreviations

EHR: electronic health record
eHealth: electronic health
IoT: Internet of Things
mHealth: mobile health
